# Invasive pulmonary aspergillosis among intubated patients with SARS-CoV-2 or influenza pneumonia: a European multicenter comparative cohort study

**DOI:** 10.1186/s13054-021-03874-1

**Published:** 2022-01-04

**Authors:** Anahita Rouzé, Elise Lemaitre, Ignacio Martin-Loeches, Pedro Povoa, Emili Diaz, Rémy Nyga, Antoni Torres, Matthieu Metzelard, Damien Du Cheyron, Fabien Lambiotte, Fabienne Tamion, Marie Labruyere, Claire Boulle Geronimi, Charles-Edouard Luyt, Martine Nyunga, Olivier Pouly, Arnaud W. Thille, Bruno Megarbane, Anastasia Saade, Eleni Magira, Jean-François Llitjos, Iliana Ioannidou, Alexandre Pierre, Jean Reignier, Denis Garot, Louis Kreitmann, Jean-Luc Baudel, Guillaume Voiriot, Gaëtan Plantefeve, Elise Morawiec, Pierre Asfar, Alexandre Boyer, Armand Mekontso-Dessap, Demosthenes Makris, Christophe Vinsonneau, Pierre-Edouard Floch, Clémence Marois, Adrian Ceccato, Antonio Artigas, Alexandre Gaudet, David Nora, Marjorie Cornu, Alain Duhamel, Julien Labreuche, Saad Nseir, the coVAPid study group, Mathilde Bouchereau, Boualem Sendid, Sean Boyd, Luis Coelho, Julien Maizel, Pierre Cuchet, Wafa Zarrougui, Déborah Boyer, Jean-Pierre Quenot, Mehdi Imouloudene, Marc Pineton de Chambrun, Thierry Van Der Linden, François Arrive, Sebastian Voicu, Elie Azoulay, Edgard Moglia, Frédéric Pene, Catia Cilloniz, Didier Thevenin, Charlotte Larrat, Laurent Argaud, Bertrand Guidet, Matthieu Turpin, Damien Contou, Alexandra Beurton, Julien Demiselle, David Meguerditchian, Keyvan Razazi, Romain Arrestier, Vassiliki Tsolaki, Mehdi Marzouk, Guillaume Brunin, Nicolas Weiss, Luis Morales

**Affiliations:** 1 CHU de Lille, Médecine Intensive-Réanimation, 59000 Lille, France; 2 INSERM U1285, CNRS, UMR 8576 – UGSF – Unité de Glycobiologie Structurale et Fonctionnelle, Université de Lille, 59000 Lille, France; 3 Department of Intensive Care Medicine, Multidisciplinary Intensive Care Research Organization (MICRO), St. James’s Hospital, Dublin, Ireland; 4 Department of Clinical medicine, School of Medicine, Trinity College Dublin, Dublin, Ireland; 5 Hospital Clinic, IDIBAPS, Universidad de Barcelona, Ciberes, Barcelona, Spain; 6 Polyvalent Intensive Care Unit, Hospital de São Francisco Xavier, CHLO, Lisbon, Portugal; 7 NOVA Medical School, CHRC, New University of Lisbon, Lisbon, Portugal; 8 Center for Clinical Epidemiology and Research Unit of Clinical Epidemiology, OUH Odense University Hospital, Odense, Denmark; 9 Critical Care Department, Hospital Universitari Parc Tauli, Sabadell, Departament de Medicina, Universitat Autonoma de Barcelona, Barcelona, Spain; 10 Service de médecine intensive réanimation, CHU Amiens Picardie, 80000 Amiens, France; 11 Department of Pulmonology, Hospital Clinic of Barcelona, IDIBAPS, CIBERES, University of Barcelona, Barcelona, Spain; 12 Department of Medical Intensive Care, Caen University Hospital, 14000 Caen, France; 13 Service de réanimation polyvalente, Centre hospitalier de Valenciennes, Valenciennes, France; 14 Medical Intensive Care Unit, UNIROUEN, Inserm U1096, FHU- REMOD-VHF, Rouen University Hospital, 76000 Rouen, France; 15 Department of Intensive Care, François Mitterrand University Hospital, Dijon, France; 16 Service de réanimation et de soins intensifs, Centre hospitalier de Douai, Douai, France; 17 Service de Médecine Intensive Réanimation, Institut de Cardiologie, Groupe Hospitalier Pitié-Salpêtrière, Assistance Publique – Hôpitaux de Paris, Paris Cedex 13, France; 18 Service de réanimation, Centre hospitalier de Roubaix, Roubaix, France; 19 Service de médecine intensive réanimation, Hôpital Saint Philibert GHICL, Université catholique, Lille, France; 20 CHU de Poitiers, Médecine Intensive Réanimation, CIC 1402 ALIVE, Université de Poitiers, Poitiers, France; 21 Department of Medical and Toxicological Critical Care, Lariboisière Hospital, INSERM UMRS-1144, Paris University, Paris, France; 22 Service de médecine intensive réanimation, Hôpital Saint-Louis, 75010 Paris, France; 23 First Department of Critical Care Medicine, Medical School, Evangelismos Hospital, National and Kapodistrian University of Athens, Athens, Greece; 24 Medical Intensive Care Unit, Cochin Hospital, Assistance Publique – Hôpitaux de Paris, Paris, France; 25 First Department of Pulmonary Medicine and Intensive Care Unit, Sotiria Chest Hospital, National and Kapodistrian University of Athens, Athens, Greece; 26 Service de réanimation polyvalente, Centre Hospitalier de Lens, Lens, France; 27 Service de Médecine Intensive Réanimation, CHU de Nantes, Nantes, France; 28 Service de Médecine Intensive Réanimation, CHU de Tours, Hôpital Bretonneau, 37044 Tours Cedex 9, France; 29 Service de Médecine Intensive - Réanimation, Hôpital Edouard Herriot, Hospices Civils de Lyon, 69437 Lyon Cedex 03, France; 30 Service de Médecine Intensive Réanimation, Hôpital Saint-Antoine, Assistance Publique-Hôpitaux de Paris, 75012 Paris, France; 31 Assistance Publique-Hôpitaux de Paris, Service de Médecine Intensive Réanimation, Hôpital Tenon, Sorbonne Université, Paris, France; 32 Service de réanimation polyvalente, CH Victor Dupouy, Argenteuil, France; 33 Service de Médecine Intensive-Réanimation et Pneumologie, Hôpital Pitié Salpêtrière, Assistance Publique-Hôpitaux de Paris, Paris, France; 34 Inserm UMRS Neurophysiologie respiratoire expérimentale et clinique, Assistance Publique-Hôpitaux de Paris, Hôpital Pitié Salpêtrière, Sorbonne Université, Paris, France; 35 Département de Médecine Intensive Réanimation, CHU d’Angers, 49933 Angers Cedex 9, France; 36 Service de médecine intensive réanimation, CHU de Bordeaux, 33000Bordeaux, France; 37 Assistance Publique-Hôpitaux de Paris, Hôpitaux Universitaires Henri-Mondor, Service de Médecine Intensive Réanimation, CARMAS ; INSERM U955, Institut Mondor de recherche Biomédicale, Université Paris Est Créteil, 94010 Créteil, France; 38 Intensive Care Unit, University Hospital of Larissa, University of Thessaly, 41110 Biopolis Larissa, Greece; 39 Intensive Care Unit, Hôpital de Béthune, 62408 Béthune, France; 40 Service de réanimation, Hôpital Duchenne, 62200 Boulogne-sur-Mer, France; 41 Assistance Publique-Hôpitaux de Paris, Sorbonne Université, Hôpital de la Pitié-Salpêtrière, Département de Neurologie, Unité de Médecine Intensive Réanimation Neurologique, Sorbonne Université, Paris, France; 42 Brain Liver Pitié-Salpêtrière (BLIPS) Study Group, INSERM UMR_S 938, Centre de recherche Saint-Antoine, Maladies métaboliques, biliaires et fibro-inflammatoire du foie, Institute of Cardiometabolism and Nutrition (ICAN), Sorbonne Université, Paris, France; 43 Intensive Care Unit, IDIBAPS, CIBERES, Hospital Universitari Sagrat Cor, Barcelona, Spain; 44 Critical Care Center, Corporacion Sanitaria Universitaria Parc Tauli, CIBER Enfermedades Respiratorias, Autonomous University of Barcelona, Sabadell, Spain; 45 CNRS, Inserm, CHU Lille, Institut Pasteur de Lille, U1019-UMR9017-CIIL-Centre d’Infection et d’Immunité de Lille, Univ. Lille, Lille, France; 46 Institut de Microbiologie, Service de Parasitologie Mycologie, CHU Lille, Pôle de Biologie-Pathologie-Génétique, 59000 Lille, France; 47 ULR 2694-METRICS : Evaluation des technologies de santé et des pratiques médicales, Univ. Lille, 59000 Lille, France; 48 Biostatistics Department, CHU de Lille, 59000 Lille, France; 49 Centre de Réanimation, CHU de Lille, 59000 Lille, France; 50 INSERM U1285, CNRS UMR 8576, Glycobiology in Fungal Pathogenesis and Clinical Applications, Université de Lille ; and Pôle de Biologie-Pathologie-Génétique, Institut de Microbiologie, Service de Parasitologie Mycologie, CHU Lille, 59000 Lille, France; 51 Department of Intensive Care Medicine, Multidisciplinary Intensive Care Research Organization (MICRO), St. James’s Hospital, Dublin, Ireland; 52 Polyvalent Intensive Care Unit, Hospital de São Francisco Xavier, CHLO, Lisbon, Portugal; 53 NOVA Medical School, CHRC, New University of Lisbon, Lisbon, Portugal; 54 Service de médecine intensive réanimation, CHU Amiens Picardie, 80000 Amiens, France; 55 Department of Medical Intensive Care, Caen University Hospital, 14000 Caen, France; 56 Service de réanimation polyvalente, Centre hospitalier de Valenciennes, Valenciennes, France; 57 Medical Intensive Care Unit, Rouen University Hospital, 76000 Rouen, France; 58 Department of Intensive Care, François Mitterrand University Hospital, Dijon, France; 59 Centre hospitalier de Douai, Service de réanimation et de soins intensifs, Douai, France; 60 Service de Médecine Intensive Réanimation, Institut de Cardiologie, Groupe Hospitalier Pitié-Salpêtrière, Assistance Publique – Hôpitaux de Paris, Paris Cedex 13, France; 61 Service de médecine intensive réanimation, Hôpital Saint Philibert GHICL, Université Catholique, Lille, France; 62 CHU de Poitiers, Médecine Intensive Réanimation, CIC 1402 ALIVE, Université de Poitiers, Poitiers, France; 63 Department of Medical and Toxicological Critical Care, Lariboisière Hospital, INSERM UMRS-1144, Paris University, Paris, France; 64 Service de médecine intensive réanimation, Hôpital Saint-Louis, 75010 Paris, France; 65 Critical Care Department, Hospital Universitari Parc Taulí, Sabadell, Spain; 66 Medical Intensive Care Unit, Cochin Hospital, Assistance Publique – Hôpitaux de Paris, Paris, France; 67 Department of Pulmonology, Hospital Clinic of Barcelona, IDIBAPS, CIBERES, University of Barcelona, Barcelona, Spain; 68 Service de réanimation polyvalente, Centre Hospitalier de Lens, Lens, France; 69 Service de Médecine Intensive Réanimation, CHU de Tours, Hôpital Bretonneau, 37044 Tours Cedex 9, France; 70 Service de Médecine Intensive - Réanimation, Hôpital Edouard Herriot, Hospices Civils de Lyon, 69437 Lyon Cedex 03, France; 71 Service de Médecine Intensive Réanimation, Hôpital Saint-Antoine, Assistance Publique-Hôpitaux de Paris, 75012 Paris, France; 72 Assistance Publique-Hôpitaux de Paris, Service de Médecine Intensive Réanimation, Hôpital Tenon, Sorbonne Université, Paris, France; 73 Service de réanimation polyvalente, CH Victor Dupouy, Argenteuil, France; 74 Service de Médecine Intensive-Réanimation et Pneumologie, Assistance Publique-Hôpitaux de Paris, Hôpital Pitié Salpêtrière, Paris, France; 75 Département de Médecine Intensive Réanimation, CHU d’Angers, 49933 Angers Cedex 9, France; 76 Service de médecine intensive réanimation, CHU de Bordeaux, 33000 Bordeaux, France; 77 Assistance Publique-Hôpitaux de Paris, Hôpitaux Universitaires Henri-Mondor, Service de Médecine Intensive Réanimation , CARMAS ; INSERM U955, Institut Mondor de recherche Biomédicale, Université Paris Est Créteil, 94010 Créteil, France; 78 Assistance Publique-Hôpitaux de Paris, Hôpitaux Universitaires Henri-Mondor, Service de Médecine Intensive Réanimation, CARMAS ; INSERM U955, Institut Mondor de recherche Biomédicale, Université Paris Est Créteil, 94010 Créteil, France; 79 Intensive Care Unit, University Hospital of Larissa, University of Thessaly, 41110 Biopolis Larissa, Greece; 80 Intensive Care Unit, Hôpital de Béthune, 62408 Béthune, France; 81 Service de réanimation, Hôpital Duchenne, 62200 Boulogne-sur-Mer, France; 82 Assistance Publique-Hôpitaux de Paris, Sorbonne Université, Hôpital de la Pitié-Salpêtrière, Département de Neurologie, Unité de Médecine Intensive Réanimation Neurologique, Sorbonne Université, Paris, France; 83 Intensive Care Unit, Hospital Universitari Sagrat Cor, Barcelona, Spain

**Keywords:** Invasive pulmonary aspergillosis, Severe influenza, COVID-19, Mechanical ventilation, Intensive care unit

## Abstract

**Background:**

Recent multicenter studies identified COVID-19 as a risk factor for invasive pulmonary aspergillosis (IPA). However, no large multicenter study has compared the incidence of IPA between COVID-19 and influenza patients.

**Objectives:**

To determine the incidence of putative IPA in critically ill SARS-CoV-2 patients, compared with influenza patients.

**Methods:**

This study was a planned ancillary analysis of the coVAPid multicenter retrospective European cohort. Consecutive adult patients requiring invasive mechanical ventilation for > 48 h for SARS-CoV-2 pneumonia or influenza pneumonia were included. The 28-day cumulative incidence of putative IPA, based on Blot definition, was the primary outcome. IPA incidence was estimated using the Kalbfleisch and Prentice method, considering extubation (dead or alive) within 28 days as competing event.

**Results:**

A total of 1047 patients were included (566 in the SARS-CoV-2 group and 481 in the influenza group). The incidence of putative IPA was lower in SARS-CoV-2 pneumonia group (14, 2.5%) than in influenza pneumonia group (29, 6%), adjusted cause-specific hazard ratio (cHR) 3.29 (95% CI 1.53–7.02, *p* = 0.0006). When putative IPA and Aspergillus respiratory tract colonization were combined, the incidence was also significantly lower in the SARS-CoV-2 group, as compared to influenza group (4.1% vs. 10.2%), adjusted cHR 3.21 (95% CI 1.88–5.46, *p* < 0.0001). In the whole study population, putative IPA was associated with significant increase in 28-day mortality rate, and length of ICU stay, compared with colonized patients, or those with no IPA or Aspergillus colonization.

**Conclusions:**

Overall, the incidence of putative IPA was low. Its incidence was significantly lower in patients with SARS-CoV-2 pneumonia than in those with influenza pneumonia.

*Clinical trial registration* The study was registered at ClinicalTrials.gov, number NCT04359693.

**Supplementary Information:**

The online version contains supplementary material available at 10.1186/s13054-021-03874-1.

## Background

Invasive pulmonary aspergillosis (IPA) was reported to be common in critically ill patients with chronic obstructive pulmonary disease (COPD) [1], acute respiratory distress syndrome (ARDS) [2], cirrhosis [3], acute hepatitis [4], or immunosuppression [5]. Previous studies also highlighted a relationship between IPA and outcomes, including mortality, duration of mechanical ventilation, and ICU length of stay [6]. Recently, critically ill patients receiving invasive mechanical ventilation for severe influenza were identified as a high-risk population for IPA [7]. Influenza-associated IPA (IAPA) was also reported to be associated with increased risk for mortality in this population.

Case series, rapidly followed by single-center and large multicenter studies, highlighted a link between COVID-19 pneumonia and IPA. The incidence of IPA ranges from 4.8 to 23% of patients with SARS-CoV-2 pneumonia receiving invasive mechanical ventilation [8–17]. Some of these studies also showed that COVID-19-associated IPA (CAPA) was associated with increased mortality and longer duration of mechanical ventilation, and ICU stay [16]. To the best of our knowledge, only one retrospective study compared the incidence of IPA between COVID-19 ARDS patients and other-viruses-related ARDS [18]. This study suggested that COVID-19 was associated with reduced incidence of IPA as compared to other ARDS patients. However, the number of included patients was limited (*n* = 172) and the study was performed in a single center.

Therefore, we conducted this planned ancillary study of the coVAPid European multicenter cohort to determine the incidence of putative IPA in SARS-CoV-2 pneumonia, compared to influenza pneumonia, in intubated critically ill patients. Secondary objectives were to determine the impact of putative IPA on morbidity and mortality, and the incidence of probable IPA, based on Verweij definition [19].

## Methods

### Study design and population

This study was a planned ancillary analysis of the coVAPid multicenter retrospective observational cohort, conducted in 36 ICUs in Europe. The methods used in the coVAPid study are described elsewhere [20]. Briefly, consecutive adult patients with SARS-CoV-2 pneumonia, influenza pneumonia, or no viral infection at ICU admission, who required invasive mechanical ventilation for more than 48 h, were included. Only patients with SARS-CoV-2 pneumonia, or influenza pneumonia, were eligible for the current ancillary study. Patients with missing data regarding the primary outcome were excluded from the current analysis.

The Ethics Committee and Institutional Review Board of the Lille University Hospital approved the study protocol (Comité de Protection des Personnes Ouest VI; approved by April 14, 2020; registration number RIPH:20.04.09.60039) as minimal-risk research using data collected for routine clinical practice and waived the requirement for informed consent. Patients (or their proxies) received written information about the study and could refuse to participate. The study was registered at ClinicalTrials.gov, number NCT04359693.

### Definitions

Blot criteria were used for IPA diagnosis, as primary outcome [21]. When at least one criterion necessary for the diagnosis of putative IPA according to Blot definition was not met, the case was classified as Aspergillus colonization. Verweij criteria were used for probable IPA diagnosis, as a secondary outcome (Additional file 1: Table E1) [19]. Suspected IPA refers to clinical suspicion associated with any positive serum or respiratory sample for Aspergillus.

### Outcomes

The primary outcome of our study was the incidence of putative IPA, according to Blot definition. The secondary outcomes included the incidence of probable IPA, according to Verweij definition; and outcomes of putative IPA, including mechanical ventilation duration, ICU length of stay, and 28-day mortality.

### Statistical analysis

Quantitative variables were expressed as median (interquartile range) and categorical variables were expressed as numbers (percentage). Patient characteristics at ICU admission and during ICU stay were described, in each group, according to aspergillosis status (none, Aspergillus colonization, and putative IPA), without formal statistical comparisons. The 28-day cumulative incidence of putative or probable IPA, or combination of colonization and putative IPA were estimated using Kalbfleisch and Prentice method, considering extubation (dead or alive) within 28 days as competing event. For the incidence of putative IPA according to Blot definition, occurrence of Aspergillus colonization was treated as a competing event, in addition to extubation [22].

Regarding the causal relationship of interest, we assessed the association of study groups with IPA (according to both definitions, as well as combining together colonization and putative IPA) using cause-specific Cox’s proportional hazard models, with sandwich covariance estimation to account for center clustering effect. We considered previously cited competing events, before and after adjustment for pre-specified confounders (simplified acute physiology score (SAPS) II, COPD, immunosuppression, recent antibiotic treatment before ICU admission, ARDS on admission, corticosteroid treatment during ICU stay) [23]. Cause-specific hazard ratios (cHR) and their 95% confidence intervals (CIs) associated with SARS-CoV-2 pneumonia, against influenza pneumonia, were derived from Cox’s models as effect sizes.

We assessed the association of putative IPA with patient’s outcomes censored at day 28 (overall survival, mechanical ventilation duration, length of ICU stay) using a Cox’s regression model (with sandwich covariance estimation to account for center clustering effect) performed on the whole study population, combining the two groups), with cause-specific hazard for mechanical ventilation duration (considering extubation alive as event of interest and death under mechanical ventilation as competing event), and for length of ICU stay (considering ICU discharge alive as event of interest, and death during ICU as competing event), including study group, IPA, and interaction between IPA status and study group. IPA was treated as a time-dependent covariate, as 3-levels categorical variable: no putative IPA or Aspergillus colonization, versus Aspergillus colonization, and putative IPA. This model accounted for exposure time of IPA, by comparing at each follow-up time event point, the current IPA status of patients who have the event to patients who are at risk (without the event of interest and without the competing event for mechanical ventilation duration and length of ICU stay). The associations were further adjusted for the same previously mentioned confounders [24].

Statistical testing was performed at the two-tailed α level of 0.05. Data were analyzed using the SAS software package, release 9.4 (SAS Institute, Cary, NC).

## Results

### Patient characteristics at ICU admission

**Fig. 1:**
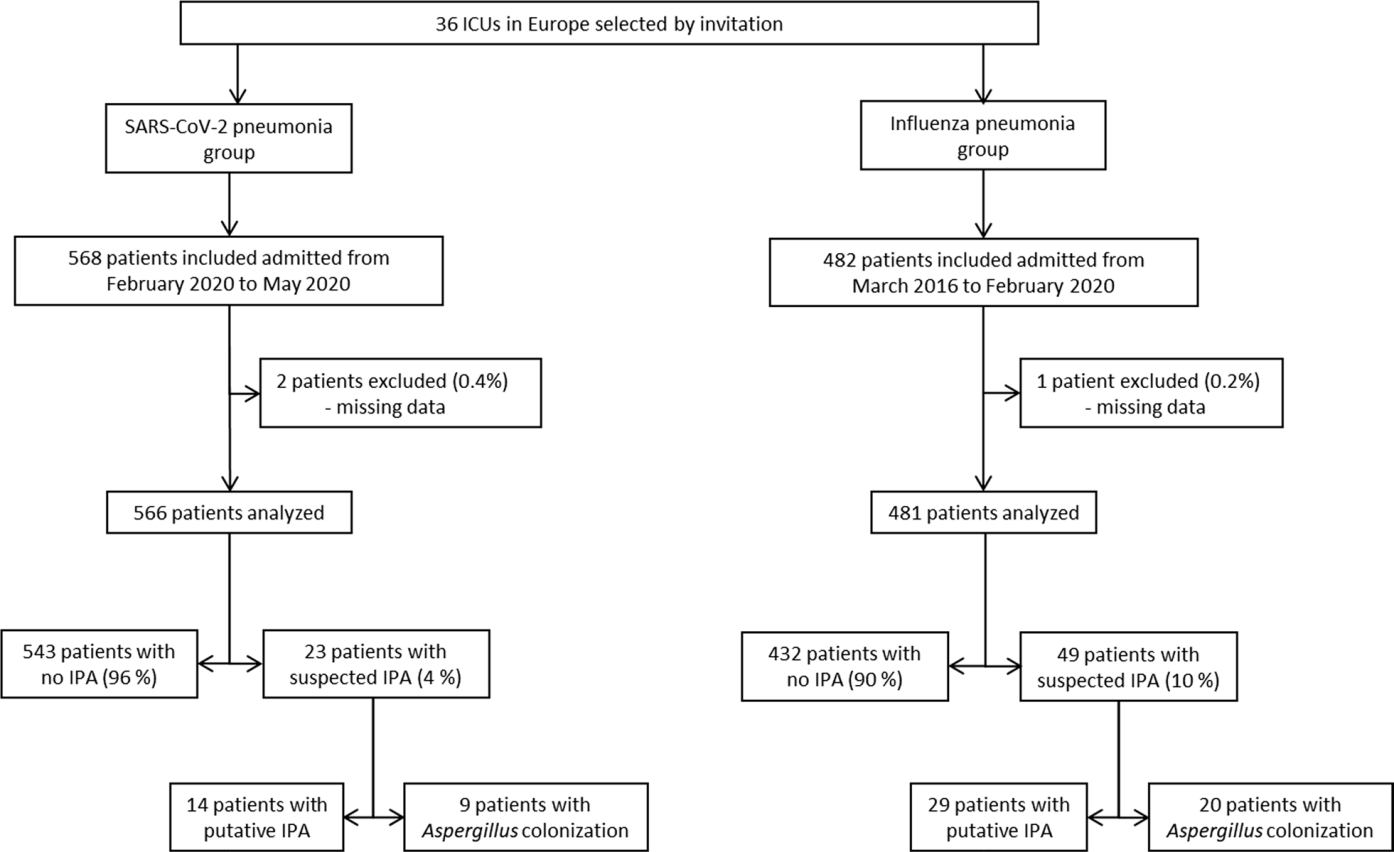
Flowchart. Suspected IPA refers to clinical suspicion associated with any positive serum or respiratory sample for Aspergillus. Putative IPA and Aspergillus colonization are defined according to Blot definition. IPA, invasive pulmonary aspergillosis

In total, 1047 patients were included (Fig. 1). Percentage of men, ARDS, and body mass index were higher in SARS-CoV-2 group than in influenza group. SAPS II, sequential organ failure assessment (SOFA) score, comorbidity scores, chronic diseases, rate of recent hospitalization, shock, cardiac arrest, neurological failure, or acute kidney injury were lower in SARS-CoV-2 pneumonia group, as compared to influenza pneumonia group (Table 1). The distribution of study patients in different centers is presented in Additional file 1: Table E3.

**Table 1:** Patient characteristics at ICU admission according to study group and aspergillosis status based on Blot definition

	SARS-CoV-2 pneumonia *n* = 566			Influenza pneumonia *n* = 481		
	No putative IPA, or colonization (*n* = 543)	Aspergillus colonization (*n* = 9)	Putative IPA (*n* = 14)	No putative IPA, or colonization (*n* = 432)	Aspergillus colonization (*n* = 20)	Putative IPA (*n* = 29)
Age, years	64 (55 to 71)	63 (62 to 68)	67 (52 to 75)	62 (53 to 71)	61 (51 to 71)	58 (52 to 64)
Men	387/543 (71.3)	8/9 (88.9)	11/14 (78.6)	271/432 (62.7)	13/20 (65.0)	14/29 (48.3)
Body mass index^†^, kg/m^2^	28.7 (25.7 to 33.6)	31.2 (26.5 to 32.5)	29.9 (28.6 to 31.8)	27.7 (23.3 to 32.7)	29.0 (25.7 to 30.4)	25.2 (21.5 to 28.5)
*Severity scores*						
SAPS II^‡^	41 (32 to 56)	44 (37 to 48)	36 (31 to 48)	50 (39 to 64)	57 (42 to 65)	47 (36 to 63)
SOFA score^§^	6 (3 to 8)	6 (5 to 9)	5 (4 to 7)	8 (6 to 11)	7 (6 to 10)	7 (4 to 12)
*Comorbidities scores*						
McCabe classification						
Non-fatal	454/518 (87.6)	8/9 (88.9)	11/14 (78.6)	288/410 (70.2)	17/18 (94.4)	19/27 (70.4)
Fatal < 5 years	58/518 (11.2)	1/9 (11.1)	3/14 (21.4)	107/410 (26.1)	1/18 (5.6)	6/27 (22.2)
Fatal < 1 year	6/518 (1.2)	0/9 (0.0)	0/14 (0.0)	15/410 (3.7)	0/18 (0.0)	2/27 (7.4)
Charlson Comorbidity Index^*ll*^	3 (1 to 4)	4 (2 to 5)	2.5 (2 to 5)	3 (2 to 5)	4 (2 to 6)	3 (1 to 4)
*Chronic diseases*						
Diabetes mellitus	159/540 (29.4)	5/9 (55.6)	4/14 (28.6)	94/425 (22.1)	4/20 (20.0)	6/28 (21.4)
Chronic kidney disease	29/535 (5.4)	3/8 (37.5)	1/14 (7.1)	35/427 (8.2)	1/20 (5.0)	3/27 (11.1)
Heart disease	98/535 (18.3)	2/9 (22.2)	2/14 (14.3)	108/426 (25.4)	3/20 (15.0)	6/29 (20.7)
Chronic heart failure	19/534 (3.6)	2/8 (25.0)	0/14 (0.0)	35/426 (8.2)	1/20 (5.0)	1/28 (3.6)
COPD	35/536 (6.5)	0/8 (0.0)	2/14 (14.3)	119/426 (27.9)	7/20 (35.0)	3/28 (10.7)
Chronic respiratory failure	19/534 (3.6)	0/8 (0.0)	1/14 (7.1)	62/426 (14.6)	2/20 (10.0)	2/28 (7.1)
Cirrhosis	8/535 (1.5)	0/8 (0.0)	0/14 (0.0)	14/426 (3.3)	1/20 (5.0)	1/28 (3.6)
Immunosuppression	46/535 (8.6)	2/8 (25.0)	2/14 (14.3)	93/429 (21.7)	2/20 (10.0)	11/29 (37.9)
Hematological malignancy	5/534 (0.9)	0/8 (0.0)	1/14 (7.1)	24/428 (5.6)	1/20 (5.0)	5/29 (17.2)
Solid cancer	25/534 (4.7)	0/8 (0.0)	0/14 (0.0)	37/428 (8.6)	1/20 (5.0)	1/29 (3.4)
Organ transplant	5/534 (0.9)	1/8 (12.5)	0/14 (0.0)	7/428 (1.6)	0/20 (0.0)	4/29 (13.8)
HIV	3/534 (0.6)	0/8 (0.0)	0/14 (0.0)	5/428 (1.2)	0/20 (0.0)	0/29 (0.0)
Immunosuppressive drugs	21/534 (3.9)	2/8 (25.0)	2/14 (14.3)	44/428 (10.3)	0/20 (0.0)	7/29 (24.1)
Active smoking	29/536 (5.4)	0/8 (0.0)	0/14 (0.0)	130/426 (30.5)	8/20 (40.0)	11/29 (37.9)
Alcohol abuse	33/534 (6.2)	1/8 (12.5)	0/14 (0.0)	75/425 (17.6)	3/20 (15.0)	7/29 (24.1)
*Location before ICU admission*						
Home	264/543 (48.6)	3/9 (33.3)	3/14 (21.4)	251/431 (58.2)	8/20 (40.0)	15/29 (51.7)
Hospital ward	199/543 (36.6)	5/9 (55.6)	11/14 (78.6)	138/431 (32.0)	7/20 (35.0)	12/29 (41.4)
Another ICU	80/543 (14.7)	1/9 (11.1)	0/14 (0.0)	42/431 (9.7)	5/20 (25.0)	2/29 (6.9)
Recent hospitalization (< 3 months)	39/541 (7.2)	2/9 (22.2)	3/14 (21.4)	61/429 (14.2)	6/20 (30.0)	5/29 (17.2)
Recent antibiotics (< 3 months)	70/542 (12.9)	1/9 (11.1)	3/14 (21.4)	79/427 (18.5)	8/20 (40.0)	7/29 (24.1)
Hospital to ICU admission, days^¥^	1 (0 to 2)	1 (0 to 2)	1 (0 to 2)	0 (0 to 1)	1 (0 to 2)	1 (0 to 4)
Hospital admission to intubation, days^¤^	1 (0 to 3)	2 (1 to 7)	2 (1 to 3)	1 (0 to 2)	1 (0 to 3)	2 (0 to 5)
Antibiotic treatment on ICU admission	475/533 (89.1)	7/9 (77.8)	12/14 (85.7)	369/421 (87.6)	19/20 (95.0)	28/29 (96.6)
*Causes for ICU admission*						
Shock	99/534 (18.5)	2/7 (28.6)	1/14 (7.1)	188/423 (44.4)	9/20 (45.0)	13/26 (50.0)
Acute respiratory failure	500/542 (92.3)	8/9 (88.9)	13/14 (92.9)	386/430 (89.8)	18/20 (90.0)	28/29 (96.6)
ARDS	370/538 (68.8)	6/9 (66.7)	8/14 (57.1)	192/422 (45.5)	13/20 (65.0)	15/26 (57.7)
Neurological failure	25/525 (4.8)	1/7 (14.3)	0/14 (0.0)	66/419 (15.8)	1/20 (5.0)	2/25 (8.0)
Cardiac arrest	3/524 (0.6)	0/7 (0.0)	0/14 (0.0)	23/419 (5.5)	0/20 (0.0)	2/25 (8.0)
Acute kidney injury	92/425 (17.5)	2/7 (28.6)	2/14 (14.3)	118/415 (28.4)	6/20 (30.0)	9/25 (36.0)

Values are as n/N (%) or median (interquartile range). ^†^100 missing values (SARS-CoV-2, *n* = 32; influenza, *n* = 68); ^‡^64 missing values (SARS-CoV-2, *n* = 43; influenza, *n* = 21); ^§^25 missing values (SARS-CoV-2, *n* = 21; influenza, *n* = 4); ^ll^30 missing values (SARS-CoV-2, *n* = 19; influenza, *n* = 11); ^¥^59 missing values (SARS-CoV-2, *n* = 31; influenza, *n* = 28); ^¤^ 75 missing values (SARS-CoV-2, n = 42; influenza, n = 33)

McCabe classification of comorbidities and likelihood of survival, likely to survive > 5 years, 1–5 years, < 1 year; Chronic kidney disease, KDOQI CKD classification stage 4 or 5 (creatinine clearance < 30 ml/mn); Chronic heart failure, NYHA class III or IV; Heart disease, ischemic heart disease or atrial fibrillation; Cirrhosis, Child–Pugh score B or C; antibiotic treatment on ICU admission, at least one dose of antibiotics in the first day of ICU stay; More than one cause for ICU admission is possible

ARDS, acute respiratory distress syndrome; COPD, chronic obstructive pulmonary disease; ICU, intensive care unit, SAPS II, simplified acute physiology score II; SOFA, sequential organ failure assessment

### Patient characteristics during ICU stay

Percentage of prone positioning, as well as total duration of antimicrobial treatment were higher in SARS-CoV-2 pneumonia group than in influenza pneumonia group. Corticosteroid use, ECMO, and 28-day mortality rates were comparable in the two groups. The dose of corticosteroids was higher in SARS-CoV-2 pneumonia group, as compared to influenza group (Table 2).

**Table 2:** Patient characteristics during ICU stay according to study group and aspergillosis status based on Blot definition

	SARS-CoV-2 pneumonia *n* = 566			Influenza pneumonia *n* = 481		
	No putative IPA, or colonization (*n* = 543)	Aspergillus colonization (*n* = 9)	Putative IPA (*n* = 14)	No putative IPA, or colonization (*n* = 432)	Aspergillus colonization (*n* = 20)	Putative IPA (*n* = 29)
Prone positioning	363/543 (66.9)	6/8 (75.0)	12/14 (85.7)	126/432 (29.2)	8/19 (42.1)	17/29 (58.6)
ECMO	58/542 (10.7)	0/9 (0.0)	2/14 (14.3)	49/432 (11.3)	5/19 (26.3)	6/28 (21.4)
Ventilator-associated lower respiratory tract infections	271/543 (49.9)	7/9 (77.8)	7/14 (50.0)	127/432 (29.4)	7/20 (35.0)	12/29 (41.4)
Antimicrobial treatment duration, days^†^	12 (7 to 18)	16 (10 to 19)	18 (8 to 20)	9 (6 to 16)	21 (12 to 28)	17 (9 to 27)
Corticosteroids	188/517 (36.4)	3/9 (33.3)	10/14 (71.4)	161/426 (37.8)	8/20 (40.0)	12/28 (42.9)
Hydrocortisone	55/512 (10.7)	2/9 (22.2)	2/14 (14.3)	92/424 (21.7)	7/20 (35.0)	7/28 (25.0)
Dexamethasone	44/512 (8.6)	0/9 (0.0)	4/14 (28.6)	1/424 (0.2)	0/20 (0.0)	0/28 (0.0)
Methylprednisolone	85/512 (16.6)	1/9 (11.1)	4/14 (28.6)	67/424 (15.8)	1/20 (5.0)	5/28 (17.9)
Highest daily dose, mg^‡^	100 (50 to 133)	50 (50 to 100)	100 (50 to 133)	50 (50 to 100)	50 (50 to 100)	63 (50 to 100)
*28-day outcomes*						
Mechanical ventilation duration, days	14 (8 to 22)	23 (12 to 28)	23 (17 to 28)	9 (5 to 18)	24 (11 to 28)	21 (12 to 28)
Ventilator-free days	6 (0 to 16)	0 (0 to 0)	1 (0 to 2)	13 (0 to 21)	1 (0 to 12)	0 (0 to 3)
ICU length of stay, days	17 (12 to 27)	28 (13 to 28)	25 (19 to 28)	13 (8 to 25)	28 (17 to 28)	25 (15 to 28)
ICU-free days	0 (0 to 12)	0 (0 to 0)	0 (0 to 0)	5 (0 to 18)	0 (0 to 2)	0 (0 to 0)
ICU mortality	154/543 (28.4)	4/9 (44.4)	5/14 (35.7)	111/432 (25.7)	3/20 (15.0)	11/29 (37.9)
28-day mortality	156/543 (28.7)	4/9 (44.4)	5/14 (35.7)	118/432 (27.3)	3/20 (15.0)	11/29 (37.9)

Values are as n/N (%) or median (interquartile range). ^†^18 missing values (SARS-CoV-2, *n* = 15; influenza, *n* = 3); ^‡^8 missing values (SARS-CoV-2, *n* = 4; influenza, *n* = 4)

Data are collected until day 28 or discharge of ICU

ECMO, extracorporeal membrane oxygenation; ICU, intensive care unit

### Incidence of putative IPA according to Blot definition

Seventy-two patients, from 25 out of 36 participating centers, were suspected by clinicians as having IPA, including 23 in SARS-CoV-2 group, and 49 in influenza group. Of these 72 patients, 43 were classified as putative IPA, and 29 as Aspergillus colonization, according to Blot definition. No proven IPA was diagnosed in study patients.

**Fig. 2:**
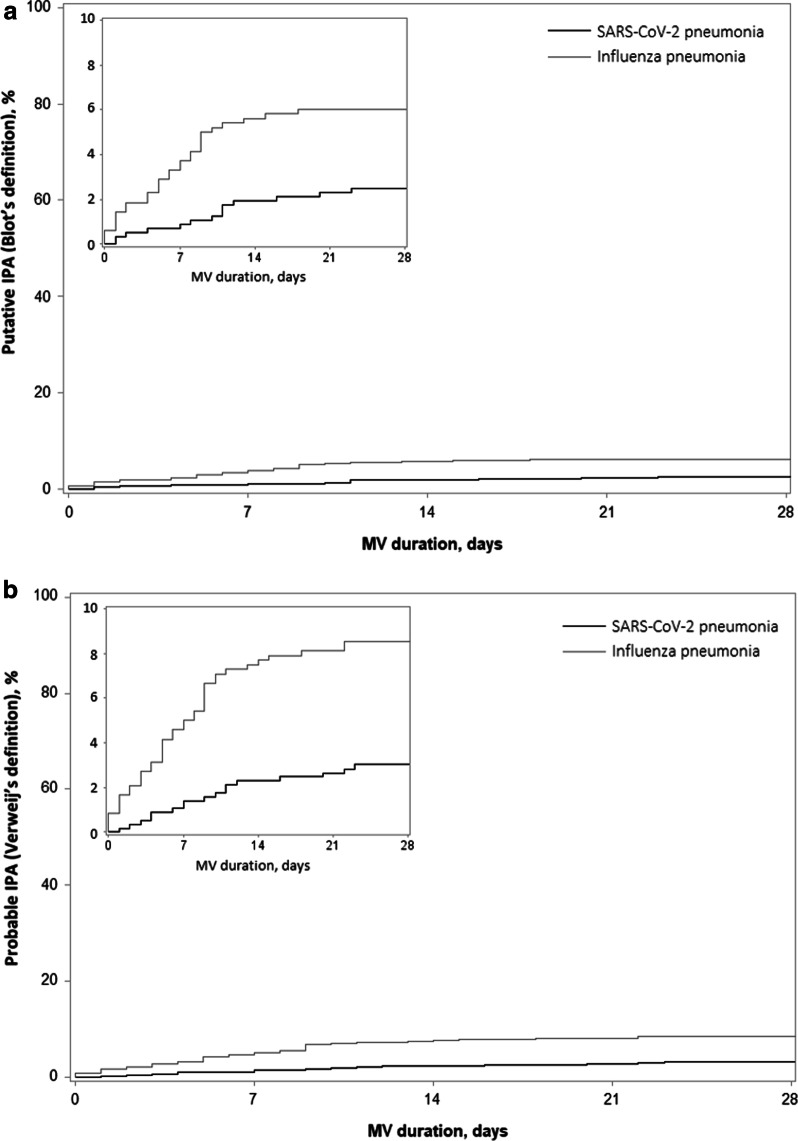
Cumulative incidence of putative or probable invasive pulmonary aspergillosis according to Blot (**A**) and Verweij (**B**) definitions. Cumulative incidence was estimated using Kalbfleisch and Prentice method, considering extubation (alive or due to death) within 28 days as competing event. Time axis starts at the day of intubation. IPA, invasive pulmonary aspergillosis, MV, mechanical ventilation

**Fig. 3:**
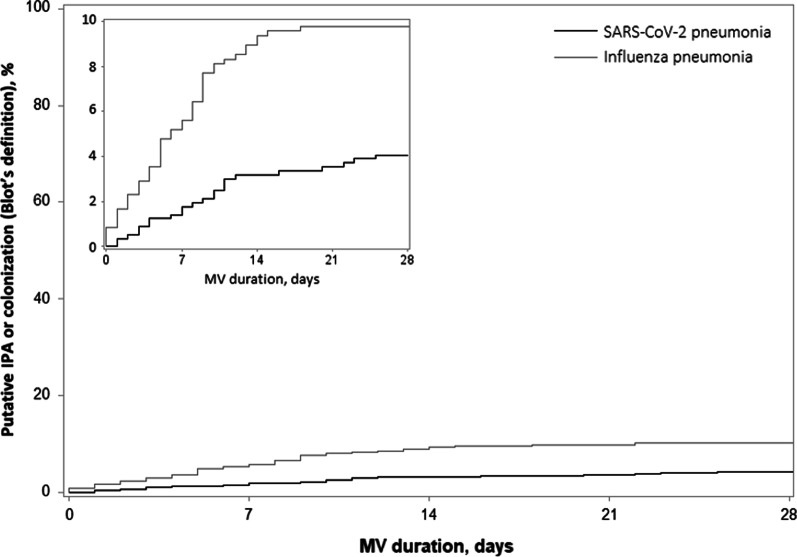
Cumulative incidence of putative invasive pulmonary aspergillosis or Aspergillus colonization according to Blot definition. Cumulative incidence was estimated using Kalbfleisch and Prentice method, considering extubation (alive or due to death) within 28 days as competing event. Time axis starts at the day of intubation. IPA, invasive pulmonary aspergillosis, MV, mechanical ventilation

The incidence of putative IPA was significantly lower in SARS-CoV-2 pneumonia group than in influenza pneumonia group (Fig. 2A, Table 3). This difference remained significant after adjustment for confounding factors. Similarly, when combining putative IPA and Aspergillus respiratory tract colonization, the incidence was still significantly lower in SARS-CoV-2 group than in influenza group (Fig. 3, Table 3). The classification of study patients, based on different definitions, is presented in Additional file 1: Table E2.

**Table 3:** Incidence of invasive pulmonary aspergillosis

	SARS-CoV-2 pneumonia *n* = 566	Influenza pneumonia *n* = 481	Unadjusted cHR (95% CI)	Adjusted cHR^*^ (95% CI)	*p* value^*^
*Blot definition*					
Putative invasive pulmonary aspergillosis	14/566 (2.5)	29/481 (6.0)	3.07 (1.52 to 6.19)	3.29 (1.53 to 7.02)	0.0006
Putative invasive pulmonary aspergillosis or Aspergillus colonization	23/566 (4.1)	49/481 (10.2)	3.17 (1.87 to 5.35)	3.21 (1.88 to 5.46)	< 0.0001
*Verweij definition*					
Probable invasive pulmonary aspergillosis	17/566 (3.0)	41/481 (8.5)	3.54 (1.86 to 6.73)	3.78 (1.96 to 7.27)	< 0.0001

Values are number of invasive pulmonary aspergillosis (28-day cumulative incidence expressed as %, considering extubation (dead or alive) as a competing event)

cHR calculated using cause-specific Cox’s proportional hazard model with sandwich covariance estimation to account for center clustering effect

^*^Adjusted for pre-specified confounders (simplified acute physiology score II, chronic obstructive pulmonary disease, immunosuppression, recent antibiotic treatment, acute respiratory distress syndrome, corticosteroid treatment), and calculated after handling missing values on covariates by multiple imputation

cHR, cause-specific hazard ratio; CI, confidence interval

### Incidence of probable IPA according to Verweij definition

Among the 72 patients suspected by physicians as having IPA, 58 patients were classified as probable IPA according to Verweij definition. The incidence of probable IPA was also significantly lower in SARS-CoV-2 group, as compared to influenza group (Fig. 2B, Table 3). This difference remained significant after adjustment for confounding factors at ICU admission.

### Outcomes of putative IPA

**Fig. 4:**
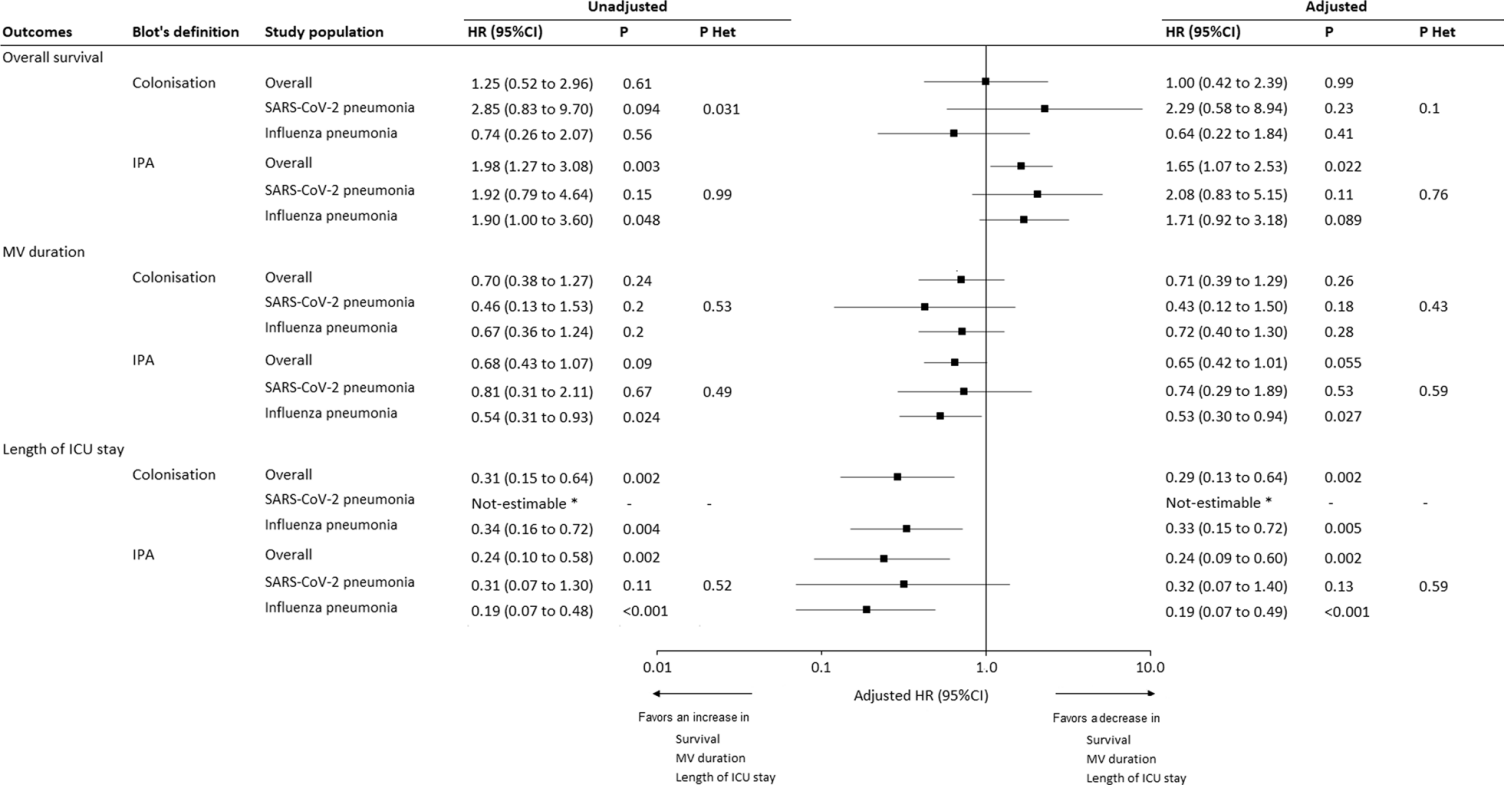
Association of putative invasive pulmonary aspergillosis, and Aspergillus colonization, according to Blot definition, with 28-day outcomes in overall population and according to study groups (SARS-CoV-2 pneumonia and influenza pneumonia). HRs were calculated using cause-specific proportional hazard models, considering death as competing event for mechanical ventilation and length of ICU stay. Adjusted HRs were calculated by including simplified acute physiology score II, chronic obstructive pulmonary disease, immunosuppression, recent antibiotic treatment before ICU admission, acute respiratory distress syndrome on admission, and corticosteroid treatment during ICU stay, as pre-specified covariates in Cox’s models (after handling missing values by multiple imputation). A HR > 1 indicates a decrease in survival (i.e., an increased risk for mortality), MV duration (i.e., an increased risk for extubation alive) and ICU length of stay (i.e., an increased risk for discharge alive) and a HR < 1 indicates an increase in survival (i.e., a decreased risk for mortality), MV duration (i.e., a decreased risk for extubation alive) and ICU length of stay (i.e., a decreased risk for discharge alive). P het indicates *p* value for heterogeneity in association of invasive pulmonary aspergillosis and 28-day outcomes across study groups (SARS-CoV-2 pneumonia vs. influenza pneumonia). * Not estimable, as no patient was discharged alive within 28 days. CI, confidence interval; HR, hazard ratio; ICU, intensive care unit; IPA, invasive pulmonary aspergillosis; MV, mechanical ventilation

In the whole study population, putative IPA was associated with significant increase in 28-day mortality rate, and length of ICU stay, compared with colonized patients, or those with no IPA or Aspergillus colonization. These results were not confirmed in the subgroups of patients with SARS-CoV-2 or influenza pneumonia. Only in influenza group, duration of mechanical ventilation, and ICU stay were significantly longer in patients with putative IPA, as compared with those with no putative IPA or Aspergillus colonization (Fig. 4).

### Characteristics of patients with putative IPA

Median time from intubation to putative IPA diagnosis was longer in SARS-CoV-2 than in influenza group (11 vs. 6 days). Bronchoalveolar lavage was less frequently performed and antifungal treatment was less frequently prescribed in SARS-CoV-2 than in influenza group (Table 4).

**Table 4:** Characteristics of patients with putative invasive pulmonary aspergillosis, according to Blot definition

	SARS-CoV-2 pneumonia *n* = 14	Influenza pneumonia *n* = 29
Time from hospital admission to IPA diagnosis	12 (7 to 14)	9 (6 to 11)
Time from ICU admission to IPA diagnosis	11 (5 to 13)	6 (2 to 10)
Time from intubation to IPA diagnosis	11 (4 to 12)	6 (2 to 9)
*Clinical presentation at the time of IPA diagnosis*		
Hemoptysis	2/14 (14.3)	4/29 (13.8)
Respiratory worsening	14/14 (100.0)	24/29 (82.8)
New or increased fever	12/14 (85.7)	15/29 (51.7)
*Imaging at the time of IPA diagnosis*		
Abnormal medical imaging (chest X-ray or CT scan)	14/14 (100.0)	29/29 (100.0)
*Predominant lesion on chest CT:*		
Dense, well-circumscribed lesion with or without a halo sign	0/5 (0.0)	3/23 (13.0)
Air-crescent sign	0/5 (0.0)	0/23 (0.0)
Cavity	0/5 (0.0)	2/23 (8.7)
Segmental or lobar consolidation	3/5 (60.0)	9/23 (39.1)
Other	2/5 (40.0)	9/23 (39.1)
*Serum samples during ICU stay*		
Galactomannan index > 0.5	6/12 (50.0)	20/26 (76.9)
Galactomannan index at the time of IPA diagnosis^†^	0.2 (0.0 to 0.6)	0.2 (0.1 to 1.4)
Highest Galactomannan index^‡^	0.2 (0.1 to 0.8)	0.5 (0.1 to 1.4)
1,3-β-D-glucan level at time of IPA diagnosis (pg/mL)^§^	63 (30 to 450)	111 (47 to 384)
Highest level of 1,3-β-D-glucan (pg/mL)^ll^	170 (39 to 760)	178 (56 to501)
*Respiratory samples leading to IPA diagnosis*		
*Type of respiratory samples:*		
Broncho-alveolar lavage	9/14 (64.3)	25/29 (86.2)
Endotracheal aspirate	7/14 (50.0)	5/29 (17.2)
Protected specimen brush	0/14 (0.0)	5/29 (17.2)
Galactomannan index ≥ 1	4/5 (80.0)	12/17 (70.6)
Galactomannan index^¥^	3.9 (2.5 to 5.6)	2.1 (0.9 to 5.8)
Positive *Aspergillus* PCR	9/12 (75.0)	11/15 (73.3)
Mycological culture	14/14 (100.0)	29/29 (100.0)
*Identified species*		
*Aspergillus fumigatus*	10/14 (71.4)	24/27 (88.9)
*Aspergillus niger*	0/14 (0.0)	1/27 (3.7)
*Aspergillus flavus*	0/14 (0.0)	1/27 (3.7)
*Aspergillus terreus*	1/14 (7.1)	1/27 (3.7)
Other species	3/14 (21.4)	0/27 (0.0)
*Antifungal treatment against aspergillosis*		
Initiation of antifungal treatment	11/14 (78.6)	27/29 (93.1)
Time from IPA diagnosis to first treatment^¤^	1 (-1 to 2)	0 (0 to 2)
*First antifungal treatment*		
Voriconazole	7/11 (63.6)	22/27 (81.5)
Isavuconazole	1/11 (9.1)	0/27 (0.0)
Caspofungin	2/11 (18.2)	2/27 (7.4)
Anidulafungin	0/11 (0.0)	1/27 (3.7)
Liposomal Amphotericin B	1/11 (9.1)	2/27 (7.4)
*Number of treatment lines used*		
1	7/14 (50.0)	17/29 (58.6)
2	3/14 (21.4)	7/29 (24.1)
3	1/14 (7.1)	3/29 (10.3)

Values are as n/N (%) or median (interquartile range). ^†^10 missing values (SARS-CoV-2, *n* = 4; influenza, *n* = 6); ^‡^5 missing values (SARS-CoV-2, *n* = 2; influenza, *n* = 3); ^§^20 missing values (SARS-CoV-2, *n* = 5; influenza, *n* = 15); ^ll^15 missing values (SARS-CoV-2, *n* = 4; influenza, *n* = 11); ^¥^22 missing values (SARS-CoV-2, *n* = 5; influenza, *n* = 17); ^¤^5 missing values (SARS-CoV-2, *n* = 3; influenza, *n* = 2)

Respiratory worsening is defined by significant PaO2/FiO2 ratio deterioration within 72 h of IPA diagnosis. New or increased fever is defined within 72 h of IPA diagnosis. All patients were intubated on the day of IPA diagnosis. More than on respiratory sample may be performed for IPA diagnosis

ICU, intensive care unit; IPA, invasive pulmonary aspergillosis; PCR, polymerase chain reaction

## Discussion

Overall, the incidence of putative IPA was low in patients with COVID-19 or influenza. Further, putative IPA incidence was significantly lower in SARS-CoV-2 pneumonia patients than in those with influenza pneumonia. Similar results were found regarding probable IPA, using Verweij definition. Putative IPA was associated with significantly higher 28-day mortality rate and length of ICU stay, compared with colonized patients, or those with no IPA or Aspergillus colonization. However, IPA was not significantly associated with increased duration of mechanical ventilation.

### Incidence of invasive pulmonary aspergillosis

The incidence of IPA was low in our study, and some previous studies reported higher incidence of IAPA and CAPA [7, 12–14, 16, 17]. However, in most of these studies, screening for IPA was performed routinely. Further, patients with no routine screening were excluded. For example, in the recent multicenter Mycovid study [16], only patients with at least 3 screening samples performed within 2 weeks were analyzed, which resulted in overestimating the reported incidence of CAPA (15%). The population at risk are all patients receiving mechanical ventilation, and not only those receiving > 2 weeks of invasive mechanical ventilation. Another potential explanation for the high incidence of IPA reported in these studies is the false positive results of galactomannan in some patients, which is supported by the absence of positive impact of antifungal treatment on mortality, and the fact that some patients with CAPA survived in spite of absence of any antifungal treatment [13]. On the other hand, other well-performed single and multicenter studies reported lower incidence of IPA in influenza and COVID-19 patients [9, 10, 18, 25], which is in line with our findings. Geographical distribution and different case definitions might explain the variation in IPA incidence.

### Comparison of invasive pulmonary aspergillosis incidence between COVID-19 and influenza patients

Our results suggest that IPA incidence might be lower in COVID-19 patients, compared with influenza patients. Several explanations could be provided for this result. First, the percentage of patients with immunosuppression at ICU admission was lower in COVID-19 than in influenza patients (8.8% vs. 22%). However, adjustment was performed for immunosuppression, as well as for other potential confounders. Second, BAL was performed less frequently in COVID-19 than in influenza patients, which might have underestimated the incidence of IPA in the first group. This could be explained by the fear of SARS-CoV-2 aerosolization and transmission to health workers at the beginning of the pandemic. Other factors, such as most severe ARDS, and more common prone position use in COVID-19 than in influenza patients could also explain the lower rate of BAL in COVID-19 patients. Third, the mechanism of entry of SARS-CoV-2, and influenza into the lower respiratory tract, and the pulmonary lesions associated with these viruses are different [26, 27]. This suggests that the lower incidence of IPA in COVID-19 patients might be specifically related to SARS-CoV-2 infection.

### Impact of invasive pulmonary aspergillosis on outcomes

In the whole study population, combining COVID-19 and influenza patients, IPA was significantly associated with increased 28-day mortality and ICU length of stay. However, the relationship between IPA and duration of mechanical ventilation did not reach significance. In subgroup analyses, IPA was associated with increased duration of mechanical ventilation and ICU length of stay in influenza, but not in COVID-19 patients. Our study is probably underpowered to determine the relationship between IPA and outcomes, or the relationship between antifungal treatment and outcomes. However, previous studies have shown a negative impact on outcome in IAPA and CAPA patients [7, 12].

### Strengths and limitations

To the best of our knowledge, our study is the first large multicenter cohort to compare the incidence of IPA between COVID-19 and influenza patients. Further, competing risk analysis, and cause-specific Cox models were used to adjust for potential confounders. However, several limitations should be acknowledged. First, the study was retrospective and there was no systematic screening for IPA, which might have underestimated the overall IPA incidence. Nevertheless, physicians prospectively identified IPA, based on clinical suspicion; and a recent taskforce recommended against routine screening for IPA in critically ill patients [23]. Second, no information was available on bronchoscopy macroscopic data, which may have also led to underestimating the incidence of IPA, because Aspergillus tracheobronchitis could not be diagnosed. Third, no information could be provided on galactomannan in some study patients, which might have also reduced the incidence of probable IPA. Fourth, the evaluation of the two diseases was not done simultaneously because of the absence of influenza during COVID-19 pandemic. Fifth, this study was conducted in Europe, mostly in France, and the results may not be generalizable to other parts of the world. Finally, we chose to use Blot definition for putative IPA, because this definition was validated using histological data in a large international study. However, galactomannan is not considered by this definition and some patients could have IPA with no Aspergillus identified in respiratory specimen. This might have also resulted in underestimating the overall incidence of IPA. However, Verweij definition was also used as a secondary outcome and although the overall IPA incidence was slightly higher in the two groups, IPA incidence was still significantly lower in COVID-19 than in influenza patients.

## Conclusions

Overall, the incidence of IPA was low in study patients. Further, putative IPA incidence was lower in SARS-COV-2 pneumonia than in influenza pneumonia patients. Our study was performed at the beginning of COVID-19 pandemic, it would be interesting to determine how IPA incidence has evolved, especially with routine use of corticosteroids in COVID-19 patients. Screening for IPA should be performed, based on recent recommendations, in patients with clinical deterioration or absence of improvement.

## Supplementary Information

**Additional file 1**. Further details on methods and results.

## Data Availability

All data needed to evaluate the conclusions in this article are present and tabulated in the main text or the appendix. This article is the result of an original retrospective cohort. For individual de-identified raw data that underlie the results reported in this article, please contact the corresponding author.

## Abbreviations

ARDS: Acute respiratory distress syndrome
CAPA: COVID-19-associated IPA
CI: Confidence interval
COPD: Chronic obstructive pulmonary disease
COVID: Coronavirus disease
HR: Hazard ratio
ICU: Intensive care unit
IPA: Invasive pulmonary aspergillosis
IAPA: Influenza-associated IPA
MV: Mechanical ventilation
SAPS II: Simplified acute physiology score II
SARS-CoV-2: Severe acute respiratory syndrome coronavirus 2
SOFA: Sequential organ failure assessment
