# Supplementary material for: Invasive pulmonary aspergillosis among intubated patients with SARS-CoV-2 or influenza pneumonia: a European multicenter comparative cohort study
Source: Crit Care. 2022 Jan 4;26:11. doi: 10.1186/s13054-021-03874-1 (PMC8724752; doi:10.1186/s13054-021-03874-1)
Supplement: Supplementary file 1 — Additional file 1. Further details on methods and results. [file 13054_2021_3874_MOESM1_ESM.docx]

**Invasive pulmonary aspergillosis among intubated patients with SARS-CoV-2 or influenza pneumonia: a European multicentre comparative cohort study**

**Online data supplement**

**METHODS**

Participating centers retrospectively collected data from consecutive patients admitted to their ICU with SARS-CoV-2 or influenza pneumonia. Centers were invited to include the same number of patients in each of the two study groups (up to 20 patients per group). Patients with confirmed SARS-CoV-2 pneumonia were included starting at the very beginning of the COVID-19 pandemic in each center. Patients with confirmed influenza A or B pneumonia were included starting from the last patients admitted with this diagnosis in 2020, and going back to previous seasons if necessary. Viral infections were confirmed by a positive result of polymerase chain reaction assay of nasopharyngeal or respiratory secretions samples.

**Statistical analysis**

Proportional hazard assumptions for each Cox’s models were assessed by examining the Schoenfeld residuals. To avoid case-deletion in multivariate analyses due to presence of missing data in pre-specified confounders, multivariable cause-specific Cox’s models were performed after handling missing data using multiple imputation procedure (1). Imputation procedure was performed using regression switching approach (chained equations with m=20 imputations obtained) under the missing at random assumption by considering all baseline characteristics, study group and outcomes (for censored outcomes by including event status and log of event time) in imputed models. Predictive mean matching method and logistic regression model (binary, ordinal or multinomial) were used for quantitative, and categorical variables; respectively. Estimates obtained in the different imputed data sets were combined using Rubin’s rules (2).

**RESULTS**

In total, 1050 patients were eligible (from March 2016 through May 2020) in the 36 participating centers (568 in the SARS-CoV-2 pneumonia group (from February 2020 through May 2020), and 482 in the influenza pneumonia group (from March 2016 through February 2020). Three patients were excluded from the analysis due to missing data (2 in the SARS-CoV-2 pneumonia group, and 1 in the influenza pneumonia group) (Figure 1).

**References**

1. Buuren S van, Groothuis-Oudshoorn. Multivariate Imputation by Chained Equations in R. J Stat Soft. J Stat Softw [Internet]. 2011 [cité 9 juin 2021];45(3). http://www.jstatsoft.org/v45/i03/

2. Gladitz J. Rubin, Donald B.: Multiple Imputation for Nonresponse in Surveys. John Wiley & Sons, Chichester – New York – Brisbane – Toronto – Singapore 1987, xxx, 258 S., 6 Abb., £ 30.25, ISSN 0271-6232. Biom J. 1989;31(1):131‑2.

| **Table E1. Definitions of putative invasive pulmonary aspergillosis, *Aspergillus* respiratory tract colonization; and probable IPA** | |
| --- | --- |
| **Putative IPA according to Blot definition** | **Probable IPA according to Verweij definition** |
| **1. Aspergillus-positive lower respiratory tract specimen culture**  **2. Compatible signs (one of the following):**  Fever refractory to at least 3 d of appropriate antibiotic therapy  Recrudescent fever after a period of defervescence of at least 48 h while still on antibiotics and without other apparent cause  Hemoptysis  Worsening respiratory insufficiency in spite of appropriate antibiotic therapy and ventilatory support  **3. Abnormal medical imaging by portable chest X-ray or CT-scan of the lungs**  **4. Either 4a or 4b:**  4a. Host risk factors (one of the following conditions) :  Underlying hematological or oncological malignancy treated with cytotoxic agents  Glucocorticoid treatment (prednisone equivalent, .20 mg/d)  Congenital or acquired immunodeficiency  4b. Semiquantitative Aspergillus-positive culture of BAL fluid positive direct microscopy | A: Pulmonary infiltrate and at least one of the following:  Serum GM index > 0.5  BAL GM index≥1.0  Positive BAL culture  ***OR***  **B**: Cavitating infiltrate (not attributed to another cause) and at least one of the following:  Positive sputum culture  Positive tracheal aspirate culture |
| ***Aspergillus* respiratory tract colonization according to Blot definition** |  |
| When at least 1 criterion necessary for the diagnosis of putative IPA is not met, the case is classified as Aspergillus colonization |  |

| **Table E2. Classifications of patients based on different definitions** | | | | | | |
| --- | --- | --- | --- | --- | --- | --- |
|  | **Physician reported** | | **Blot definition** | | **Verweij definition** | |
|  | CAPA  (n = 23) | IAPA  (n = 49) | CAPA  (n = 14) | IAPA  (n = 29) | CAPA  (n = 17) | IAPA  (n = 41) |
| **Physician reported** |  |  |  |  |  |  |
| CAPA (n = 23) |  |  | 14 |  | 17 |  |
| IAPA (n = 49) |  |  |  | 29 |  | 41 |
| **Blot definition** |  |  |  |  |  |  |
| CAPA (n = 14) | 14 |  |  |  | 10 |  |
| IAPA (n = 29) |  | 29 |  |  |  | 29 |
| **Verweij definition** |  |  |  |  |  |  |
| CAPA (n = 17) | 17 |  | 10 |  |  |  |
| IAPA (n = 41) |  | 41 |  | 29 |  |  |

**Table E3. Distribution of putative IPA in different study centers.**

| Center N° | Country | SARS-CoV-2 | | Influenza | |
| --- | --- | --- | --- | --- | --- |
|  |  | **Putative IPA**  **n (%)** | **Aspergillus colonization**  **n (%)** | **Putative IPA**  **n (%)** | **Aspergillus colonization**  **n (%)** |
| 1 | France | 1/86 (1.2) | 2/86 (2.3) | 3/80 (3.8) | 7/80 (8.8) |
| 2 | France | 0/10 (0) | 0/10 (0) | 0/8 (0) | 1/8 (12.5) |
| 3 | France | 0/9 (0) | 0/9 (0) | 0/5 (0) | 0/5 (0) |
| 4 | France | 0/15 (0) | 0/15 (0) | 0/18 (0) | 1/18 (5.6) |
| 5 | France | 1/32 (3) | 0/32 (0) | 0/18 (0) | 3/18 (16.7) |
| 6 | France | 1/20 (5) | 0/20 (0) | 2/22 (9.1) | 1/22 (4.5) |
| 7 | France | 0/10 (0) | 0/10 (0) | 1/9 (11.1) | 0/9 (0.0) |
| 8 | France | 1/10 (10) | 0/10 (0) | 1/10 (10) | 0/10 (0) |
| 9 | France | 0/10 (0) | 0/10 (0) | 0/10 (0) | 0/10 (0) |
| 10 | France | 3/20 (15) | 0/20 (0) | 2/20 (10) | 0/20 (0) |
| 11 | France | 0/10 (0) | 1/10 (10) | 0/10 (0) | 0/10 (0) |
| 12 | France | 0/10 (0) | 0/10 (0) | 0/10 (0) | 0/10 (0) |
| 13 | France | 0/10 (0) | 0/10 (0) | 1/10 (10) | 0/10 (0) |
| 14 | France | 0/10 (0) | 0/10 (0) | 2/11 (18.2) | 0/11 (0) |
| 15 | France | 0/20 (0) | 0/20 (0) | 0/7 (0) | 0/7 (0) |
| 16 | France | 0/10 (0) | 0/10 (0) | 0/9 (0) | 0/9 (0) |
| 17 | France | 0/12 (0) | 1/12 (8.3) | 0/12 (0) | 1/12 (8.3) |
| 18 | France | 3/12 (25) | 1/12(8.3) | 0/11 (0) | 0/11 (0) |
| 19 | France | 0/10 (0) | 1/10 (10) | 0/3 (0) | 0/3 (0) |
| 20 | France | 0/20 (0) | 0/20 (0) | 2/20 (10) | 1/20 (5) |
| 21 | France | 0/17 (0) | 0/17 (0) | 0/8 (0) | 0/8 (0) |
| 22 | France | 2/19 (10.5) | 0/19 (0) | 2/5 (40) | 0/5 (0) |
| 23 | France | 0/10 (0) | 0/10 (0) | 2/10 (20) | 0/10 (0) |
| 24 | France | 0/22 (0) | 0/22 (0) | 3/22 (13.6) | 0/22 (0) |
| 25 | France | 0/10 (0) | 0/10 (0) | 0/10 (0) | 0/10 (0) |
| 26 | France | 0/10 (0) | 2/10 (20) | 1/10 (10) | 0/10 (0) |
| 27 | France | 2/20 (10) | 0/20 (0) | 0/20 (0) | 1/20 (5) |
| 28 | France | 0/10 (0) | 1/10 (0) | 2/10 (20) | 0/10 (0) |
| 29 | Ireland | 0/25 (0) | 0/25 (0) | 2/25 (8) | 2/25 (8) |
| 30 | Portugal | 0/19 (0) | 0/19 (0) | 0/11 (0) | 0/11 (0) |
| 31 | Spain | 0/10 (0) | 0/10 (0) | 0/10 (0) | 0/10 (0) |
| 32 | Spain | 0/10 (0) | 0/10 (0) | 1/10 (10) | 0/10 (0) |
| 33 | Spain | 0/10 (0) | 0/10 (0) | 1/10 (10) | 1/10 (10) |
| 34 | Greece | 0/11 (0) | 0/11 (0) | 1/10 (10) | 1/10 (10) |
| 35 | Greece | 0/10 (0) | 0/10 (0) | 0/10 (0) | 0/10 (0) |
| 36 | Greece | 0/9 (0) | 0/9 (0) | 0/8 (0) | 0/8 (0) |
